# Supplementary figures and images for: A comparative study of the capacity of mesenchymal stromal cell lines to form spheroids
Source: PLoS One. 2020 Jun 2;15(6):e0225485. doi: 10.1371/journal.pone.0225485 (PMC7266346; doi:10.1371/journal.pone.0225485)

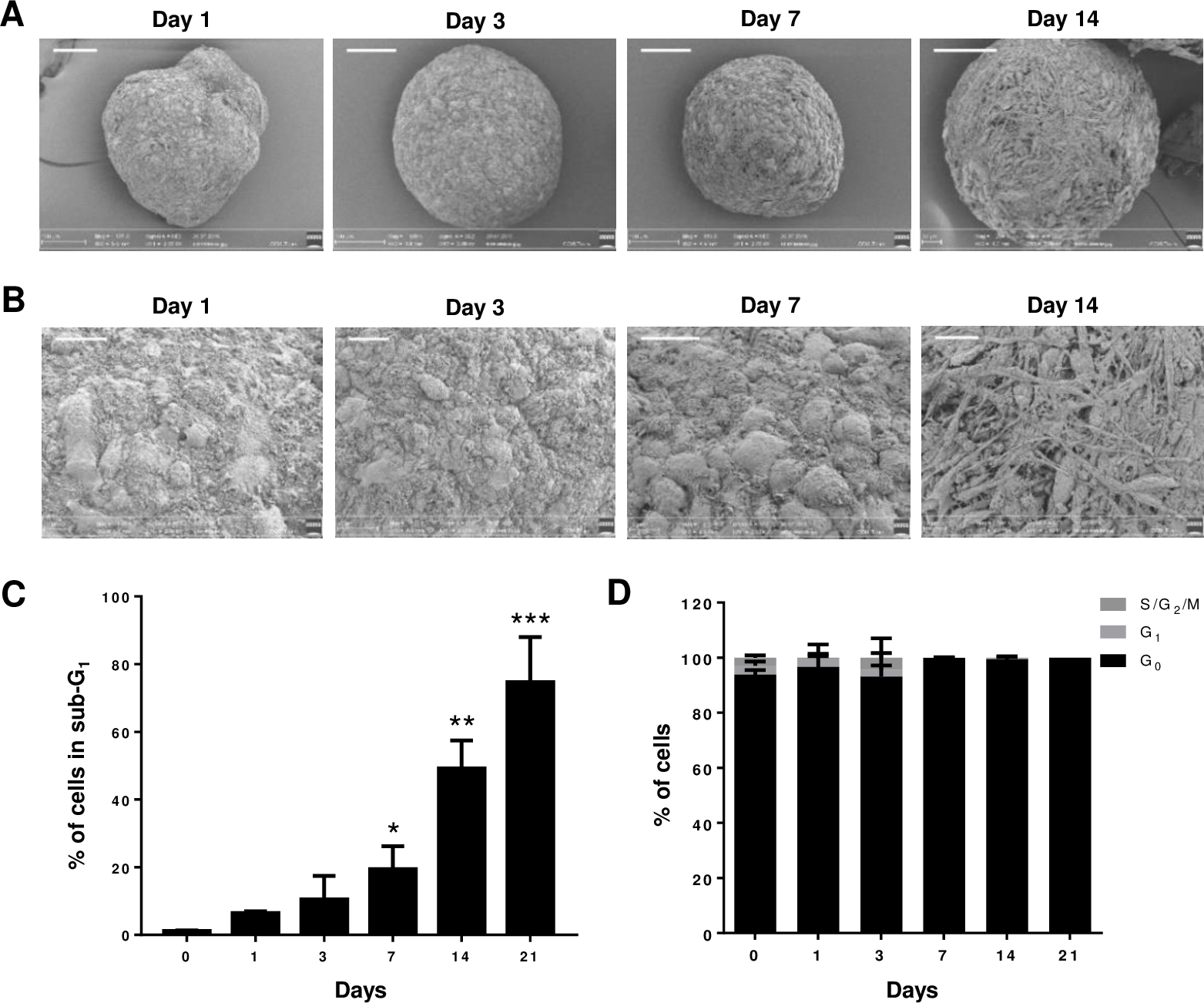

Supplement: S1 Fig — (A and B) Scanning electron microscopy (SEM) analysis over 14 days (scale bars = 100 μm (A) and 20 μm (B). (C) Sub-G1 apoptosis quantification (n = 3) and (D) cell cycle quantification over 21 days in culture (n = 3; data are mean ± SD). (TIF) [file pone.0225485.s001.tif]

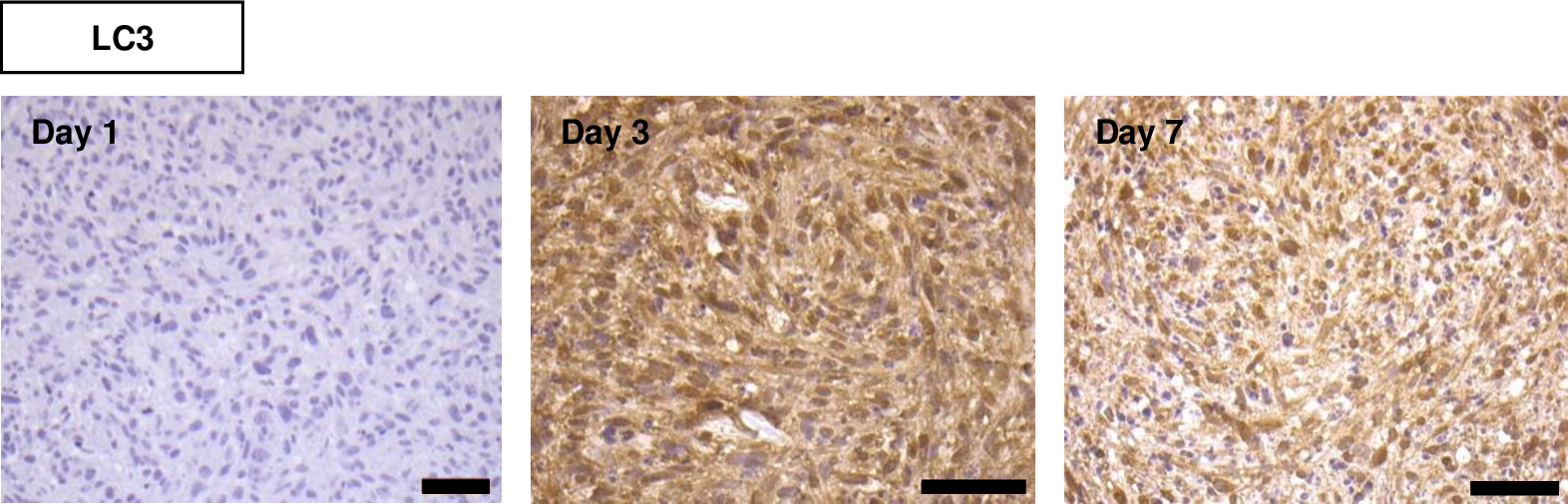

Supplement: S3 Fig — Immunohistochemistry of LC3B is shown at days 1, 3 and 7 for HS-27a-spheroids (scale bars = 50 μm). (TIF) [file pone.0225485.s003.tif]
