# Supplementary material for: A comparative study of the capacity of mesenchymal stromal cell lines to form spheroids
Source: PLoS One. 2020 Jun 2;15(6):e0225485. doi: 10.1371/journal.pone.0225485 (PMC7266346; doi:10.1371/journal.pone.0225485)
Supplement: S2 Fig — TEM analysis of primary hMSC-spheroids at day 1 (A), day 3 (B) and day 7 (C); Higher magnification is also shown to highlight autophagosomes. HS-27a-spheroids at day 1 (D), day 3 (E) and day 7 (F); HS-5-spheroids at day 1 (G), day 3 (H) and day 7 (I) and MS-5-spheroids at day 1 (J), day 3 (K) and day 7 (L). Scale bars = 20 μm. (PPTX) [file pone.0225485.s002.pptx]

## Slide 1
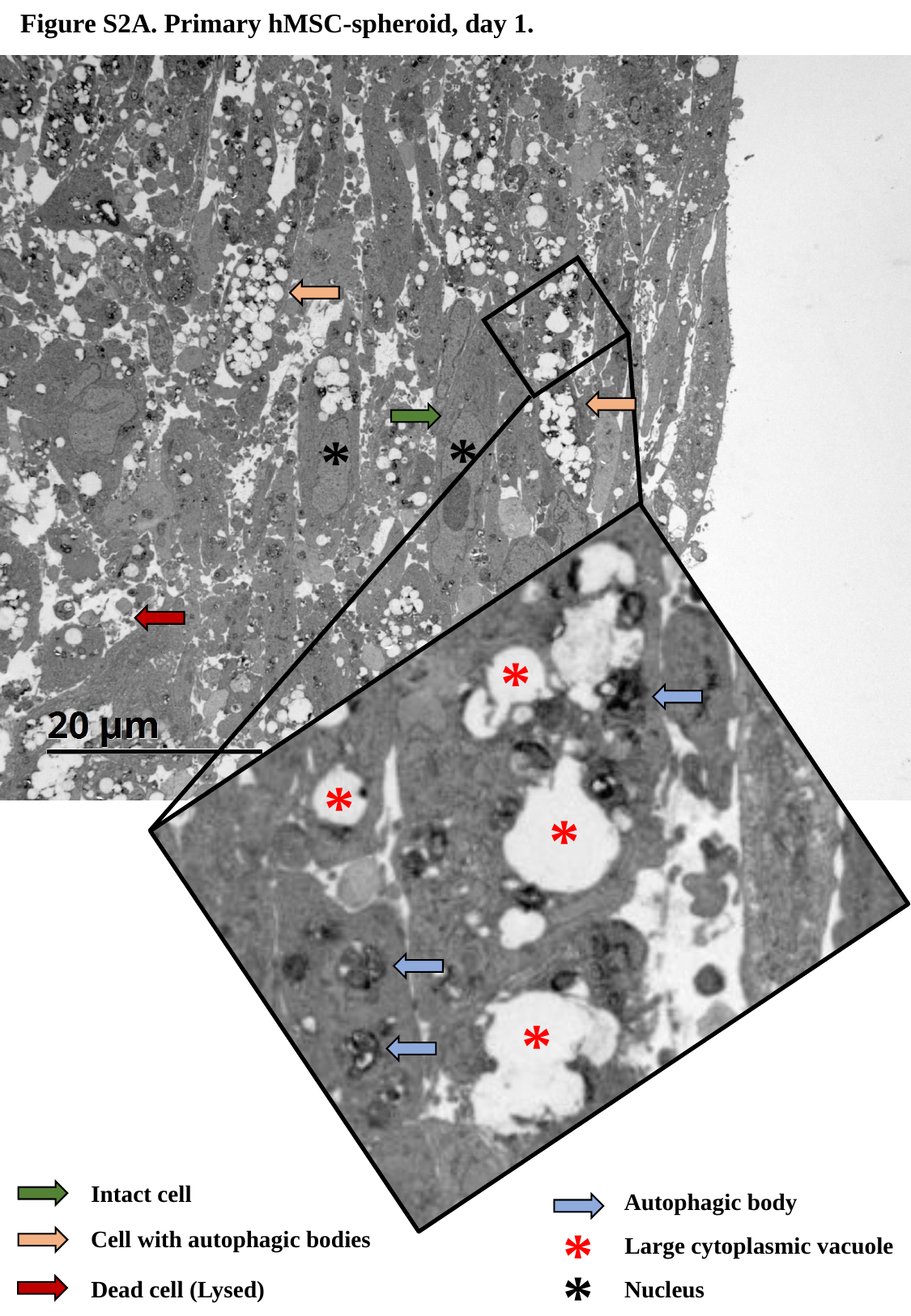

Figure S2A. Primary hMSC-spheroid, day 1.
*
*
*
*
*
*
Intact cell
Autophagic body
*
Cell with autophagic bodies
Large cytoplasmic vacuole
*
Nucleus
Dead cell (Lysed)

## Slide 2
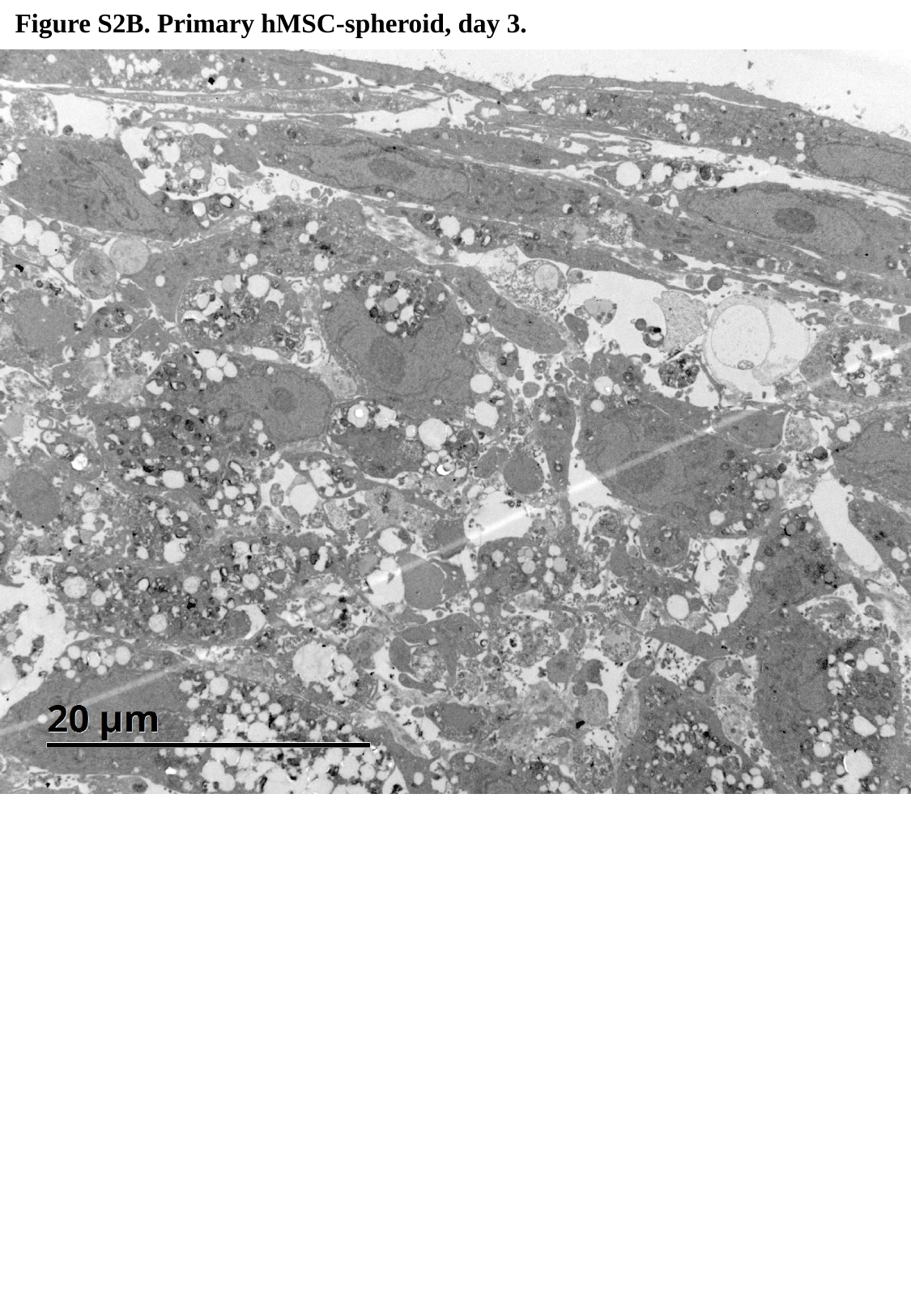

Figure S2B. Primary hMSC-spheroid, day 3.

## Slide 3
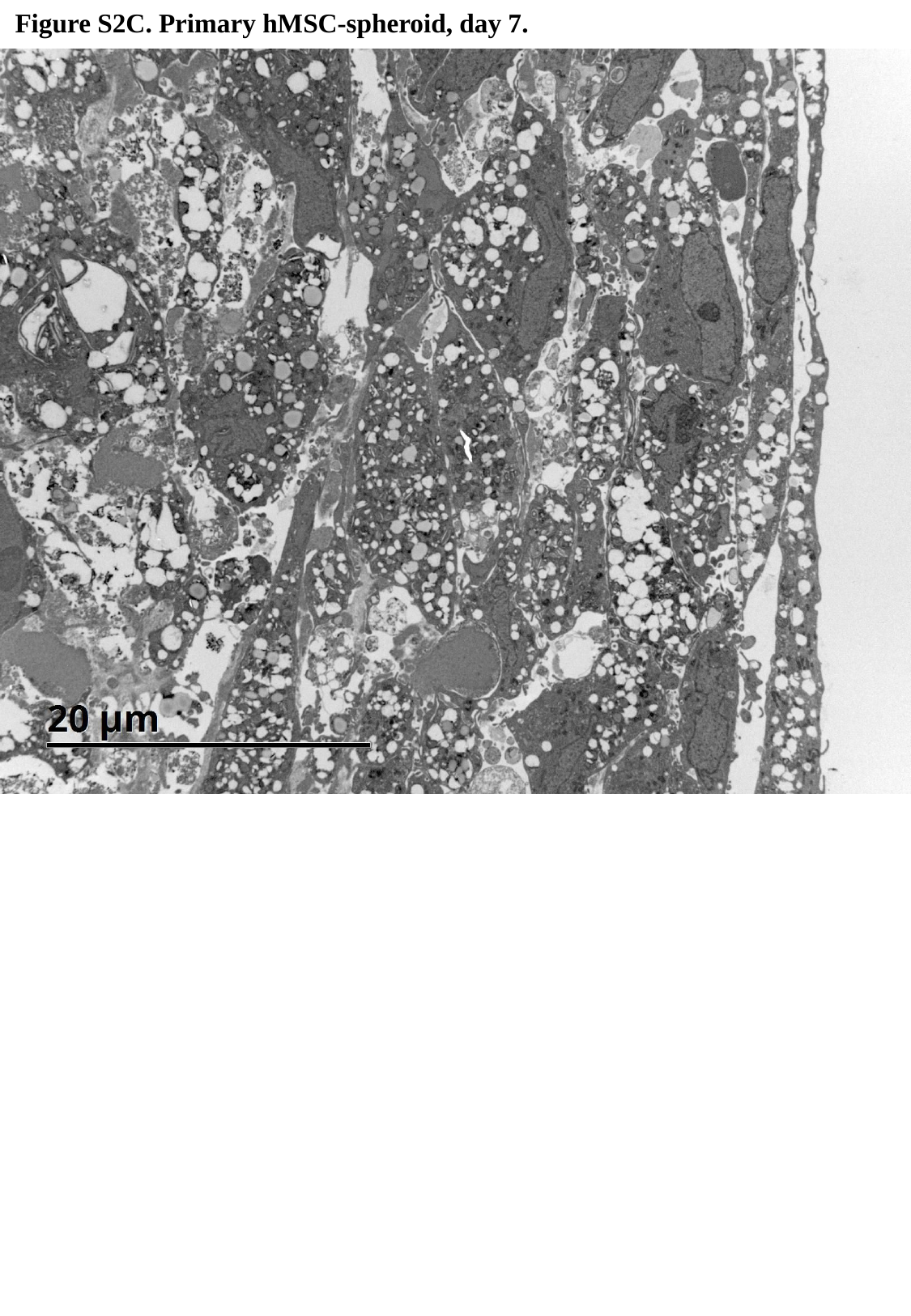

Figure S2C. Primary hMSC-spheroid, day 7.

## Slide 4
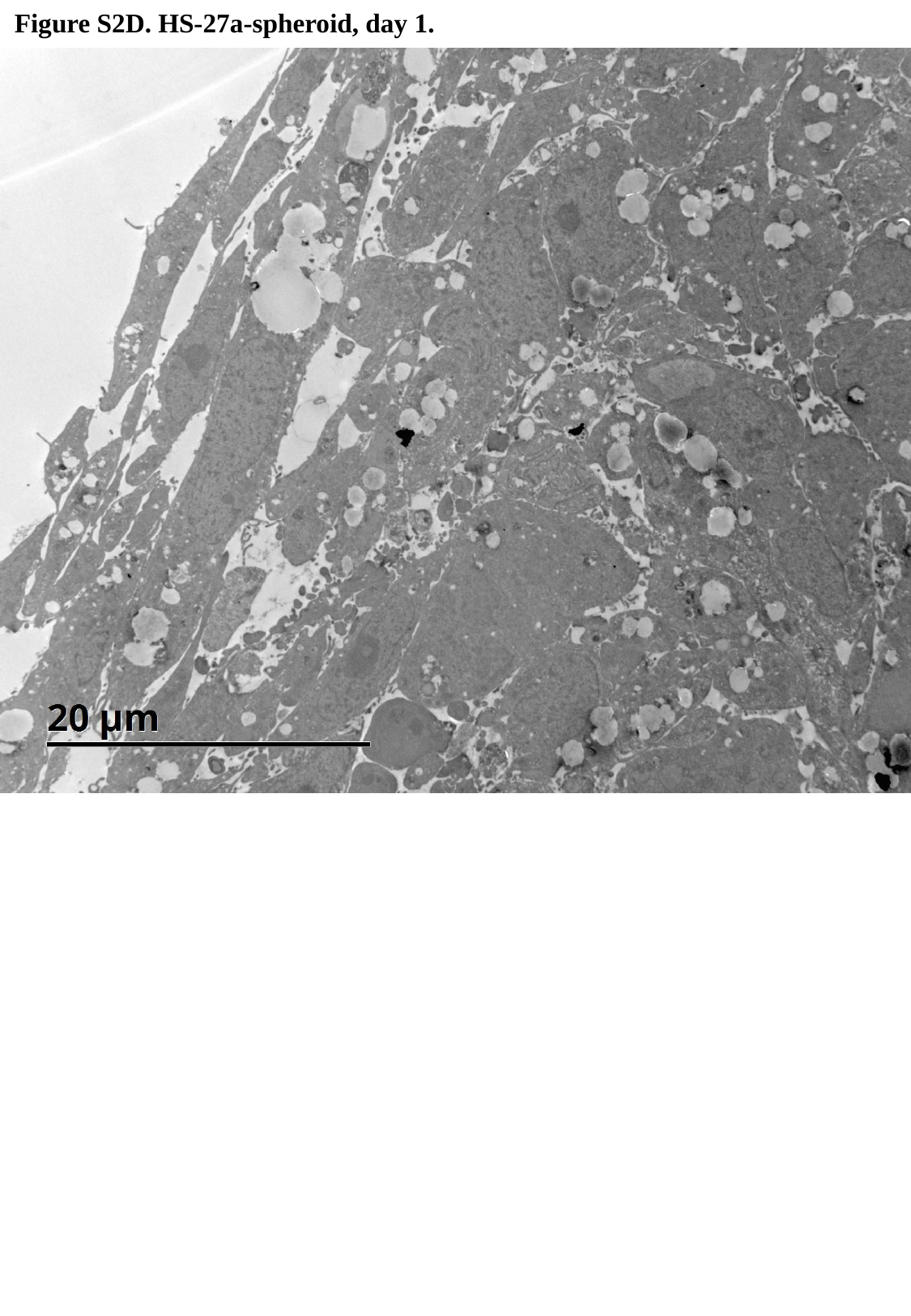

Figure S2D. HS-27a-spheroid, day 1.

## Slide 5
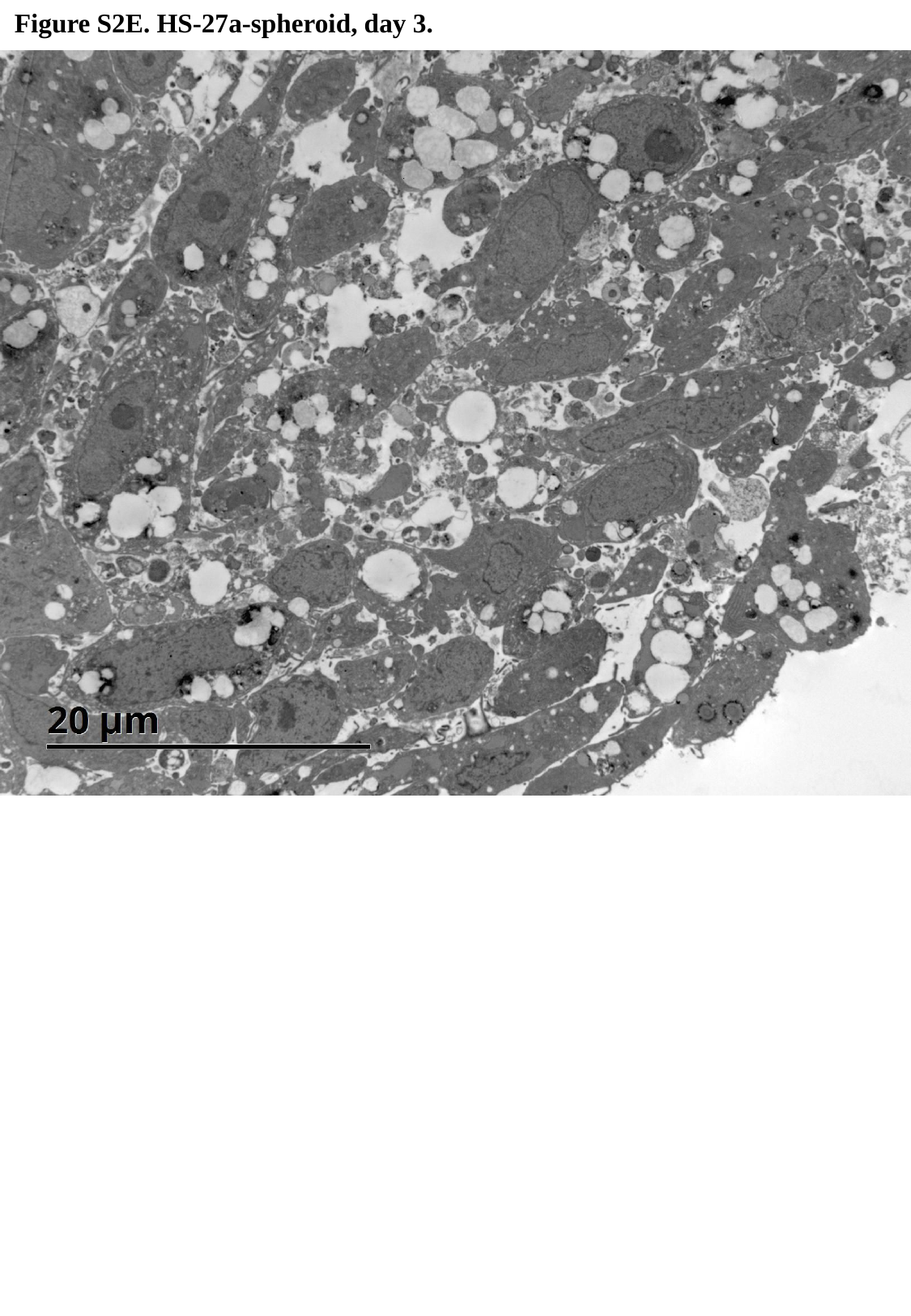

Figure S2E. HS-27a-spheroid, day 3.

## Slide 6
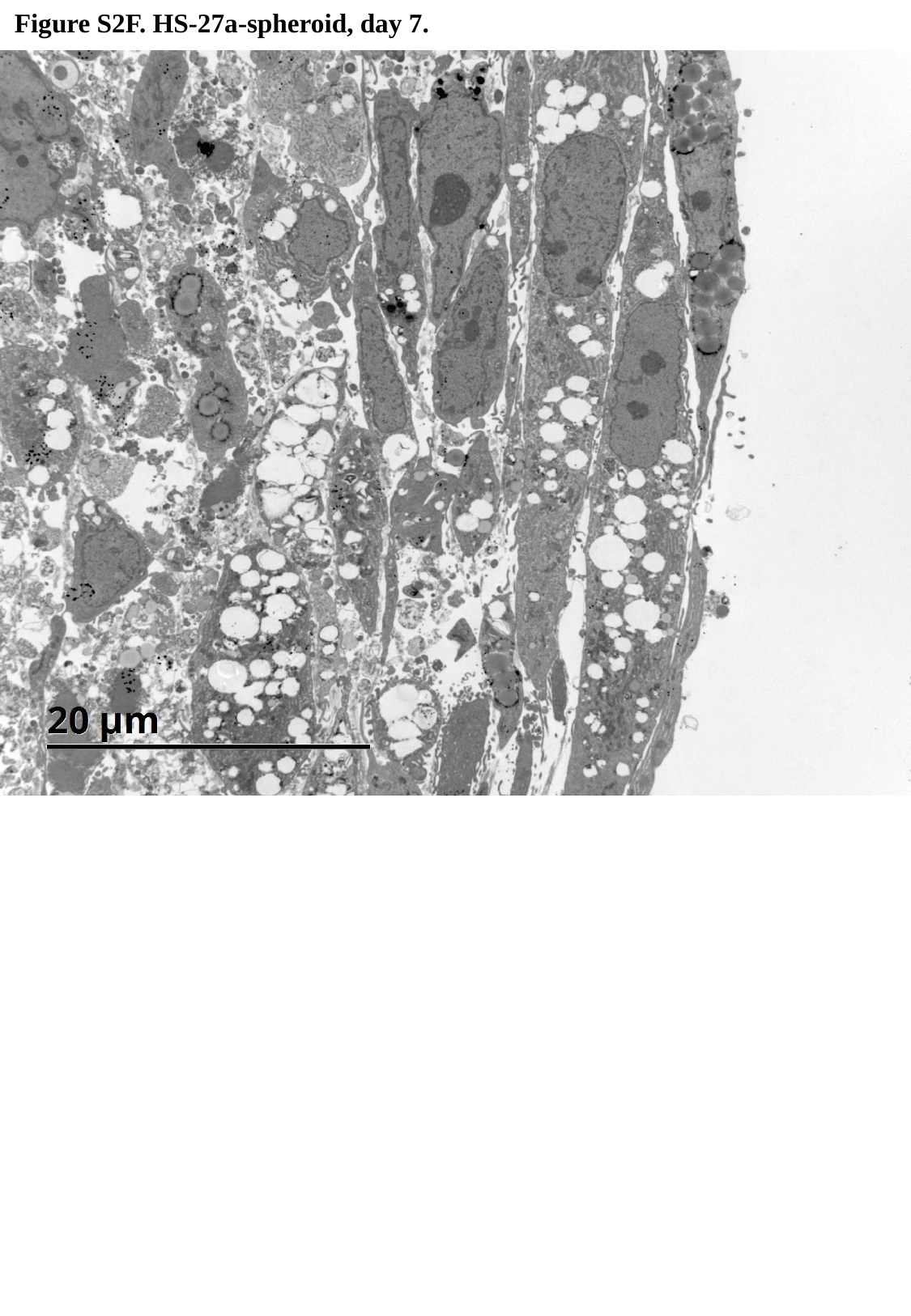

Figure S2F. HS-27a-spheroid, day 7.

## Slide 7
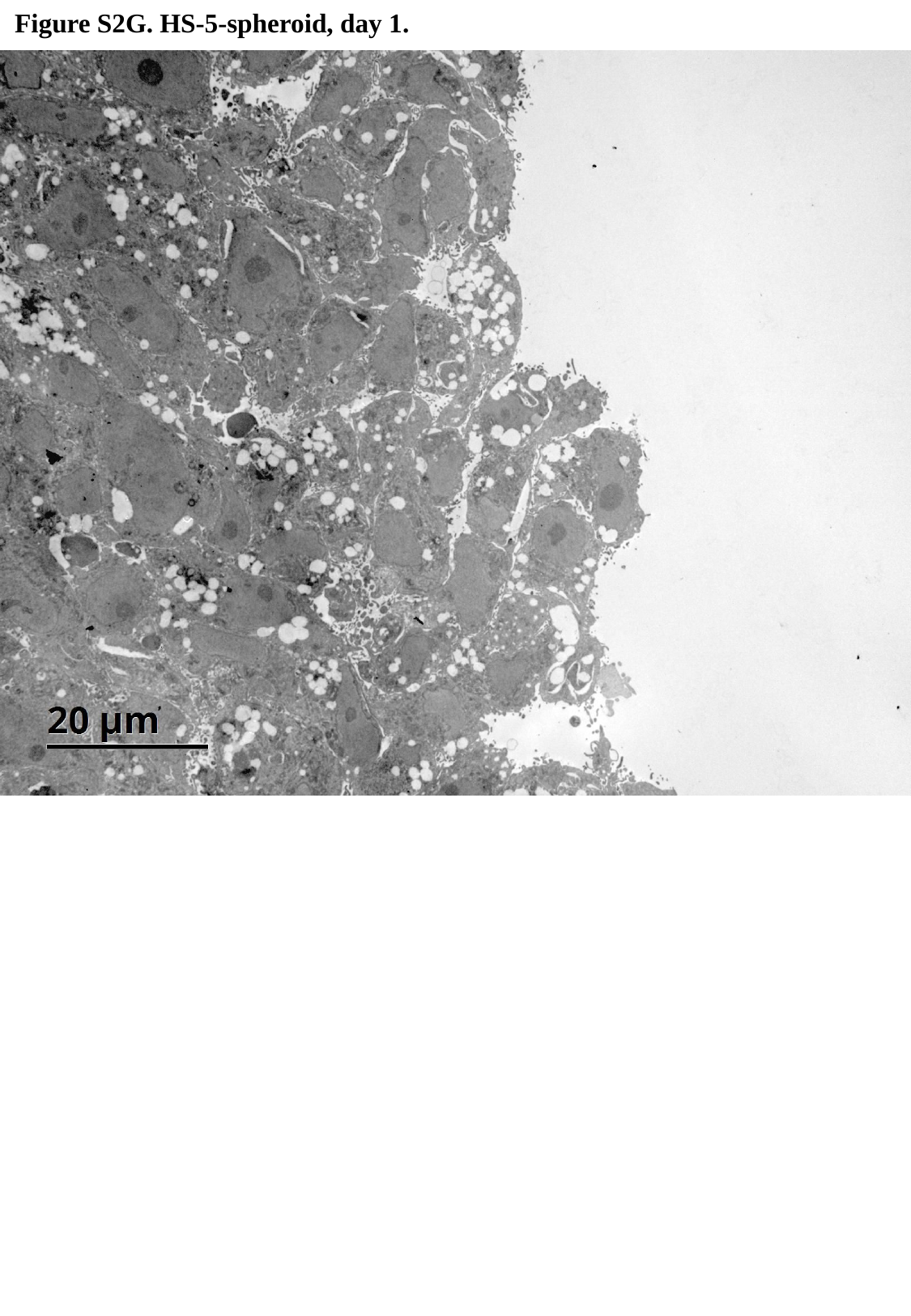

Figure S2G. HS-5-spheroid, day 1.

## Slide 8
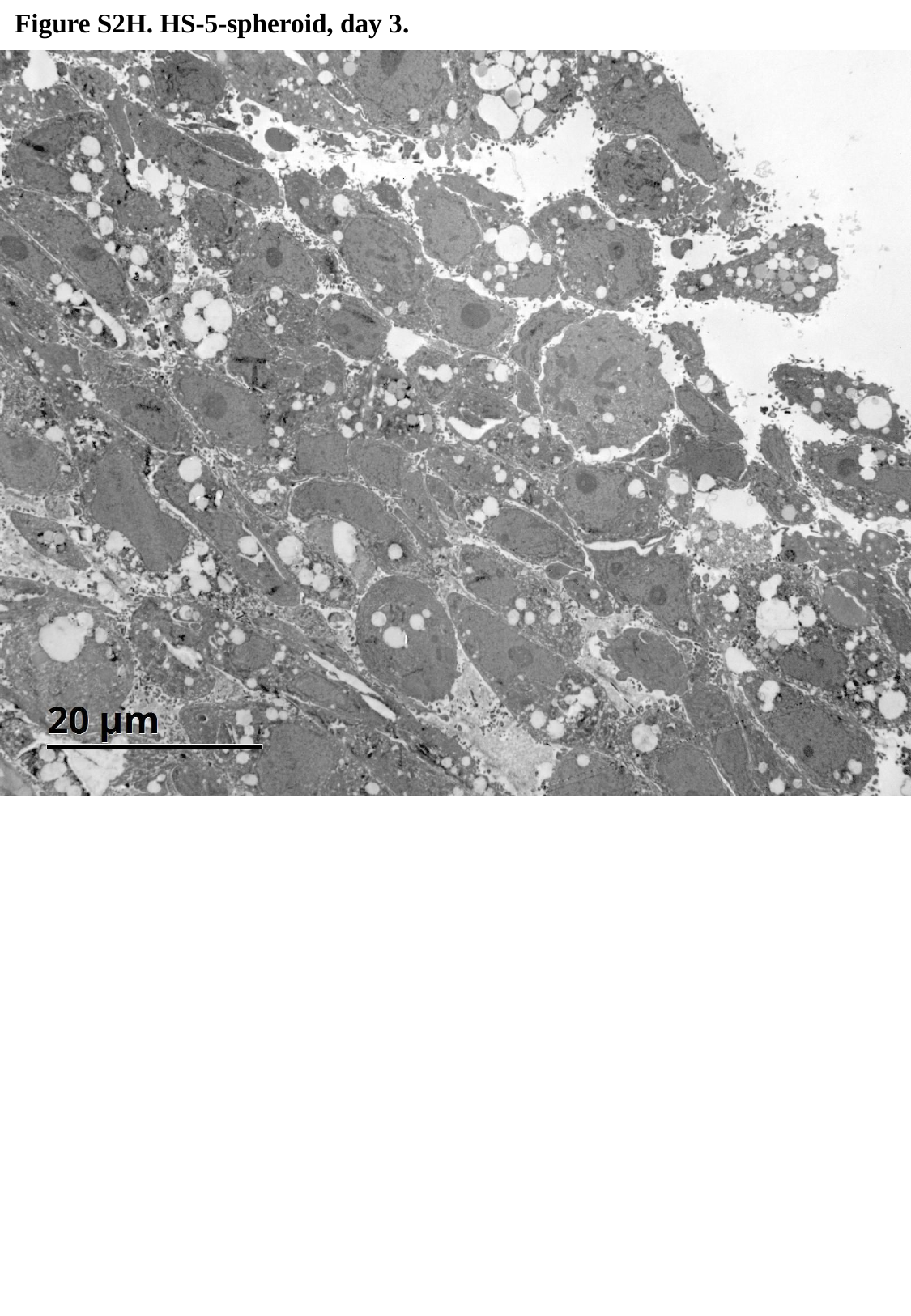

Figure S2H. HS-5-spheroid, day 3.

## Slide 9
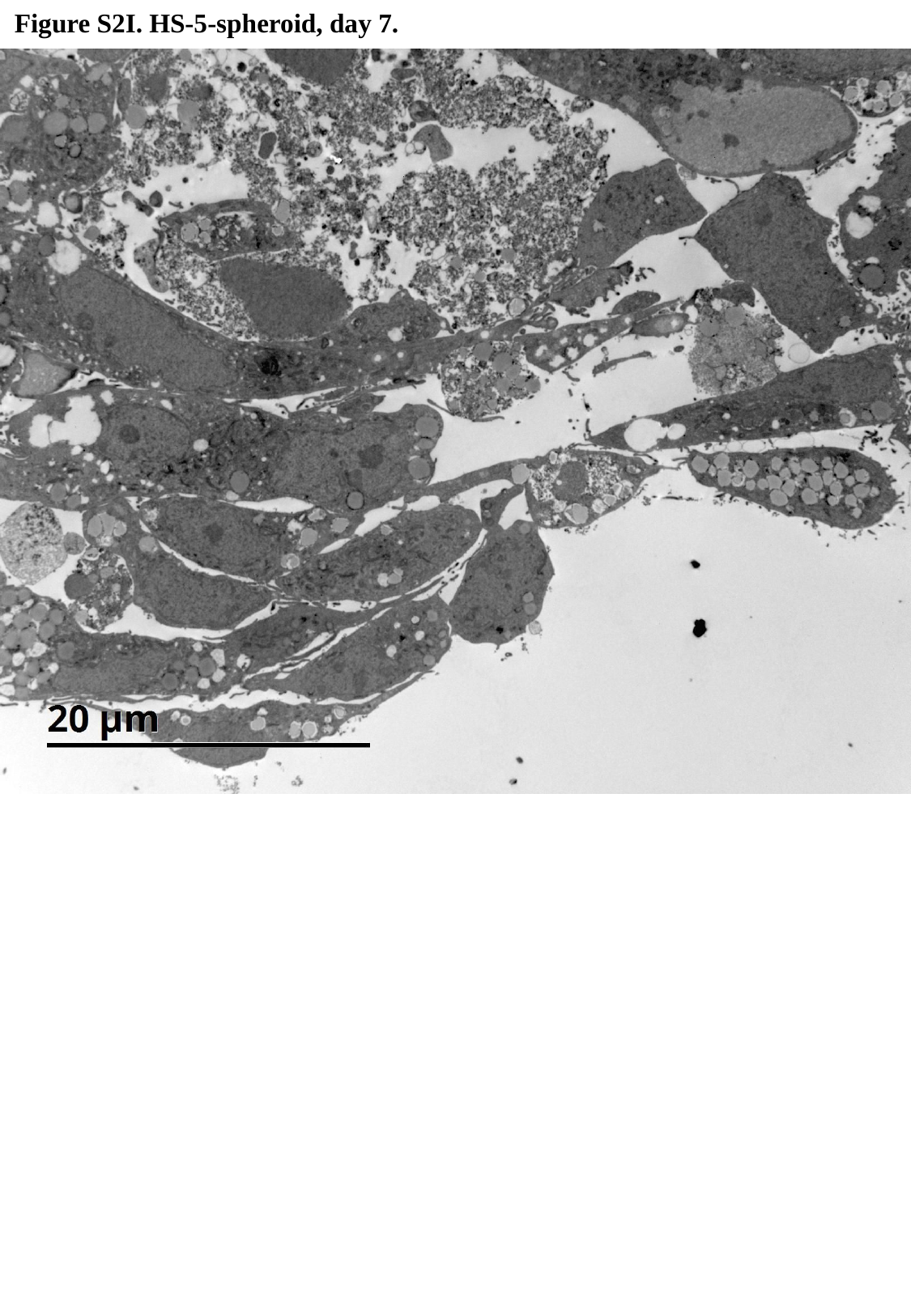

Figure S2I. HS-5-spheroid, day 7.

## Slide 10
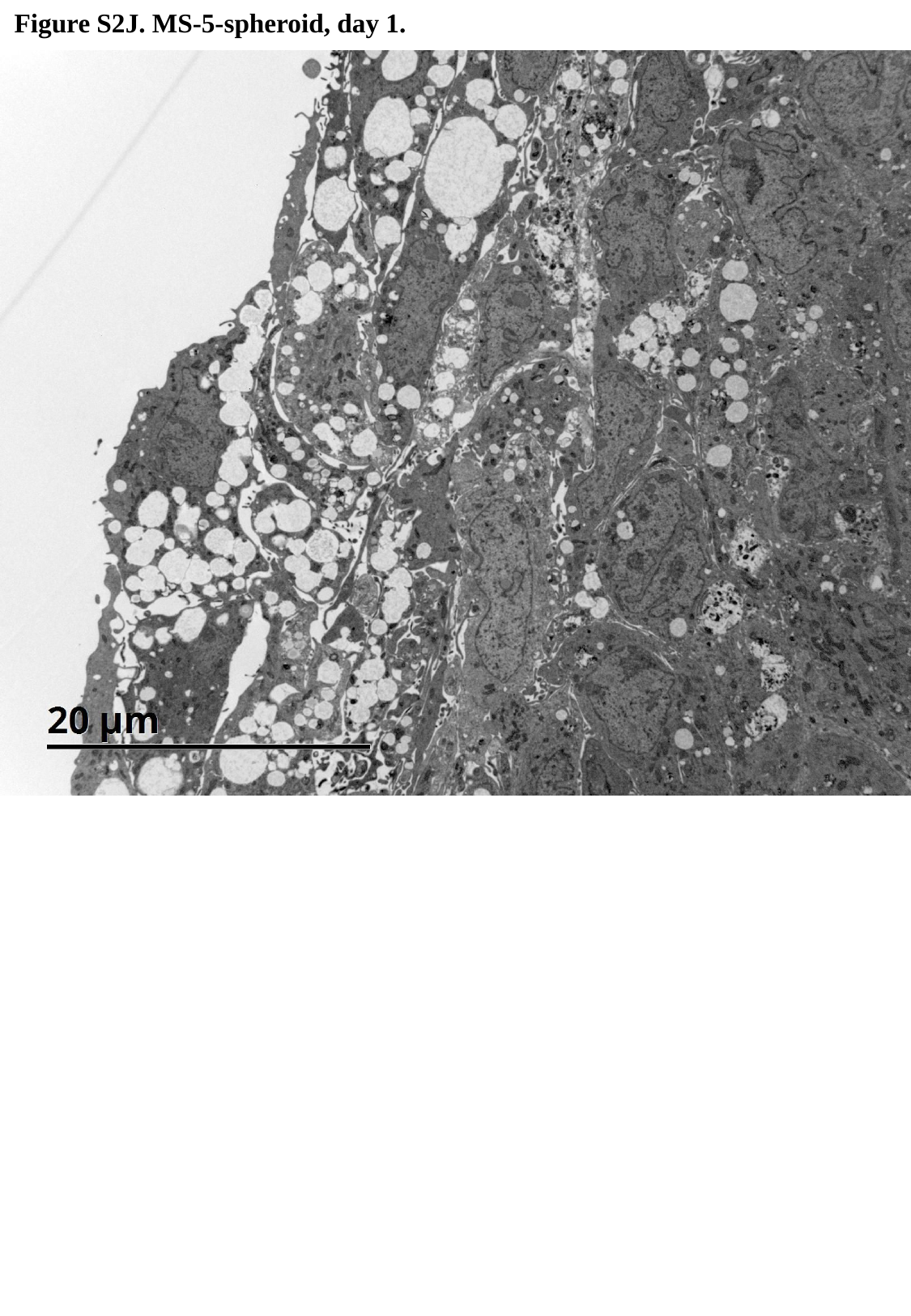

Figure S2J. MS-5-spheroid, day 1.

## Slide 11
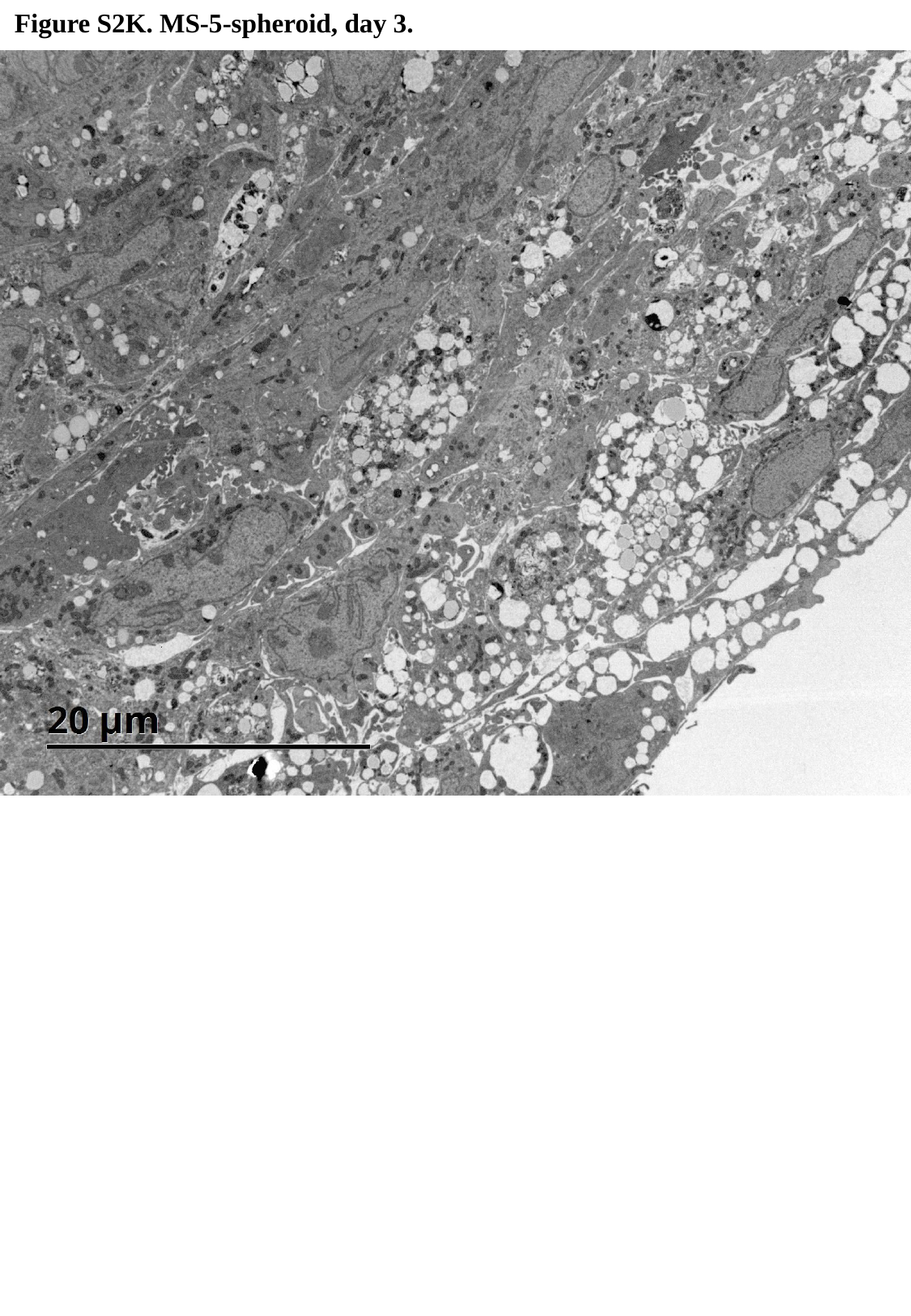

Figure S2K. MS-5-spheroid, day 3.

## Slide 12
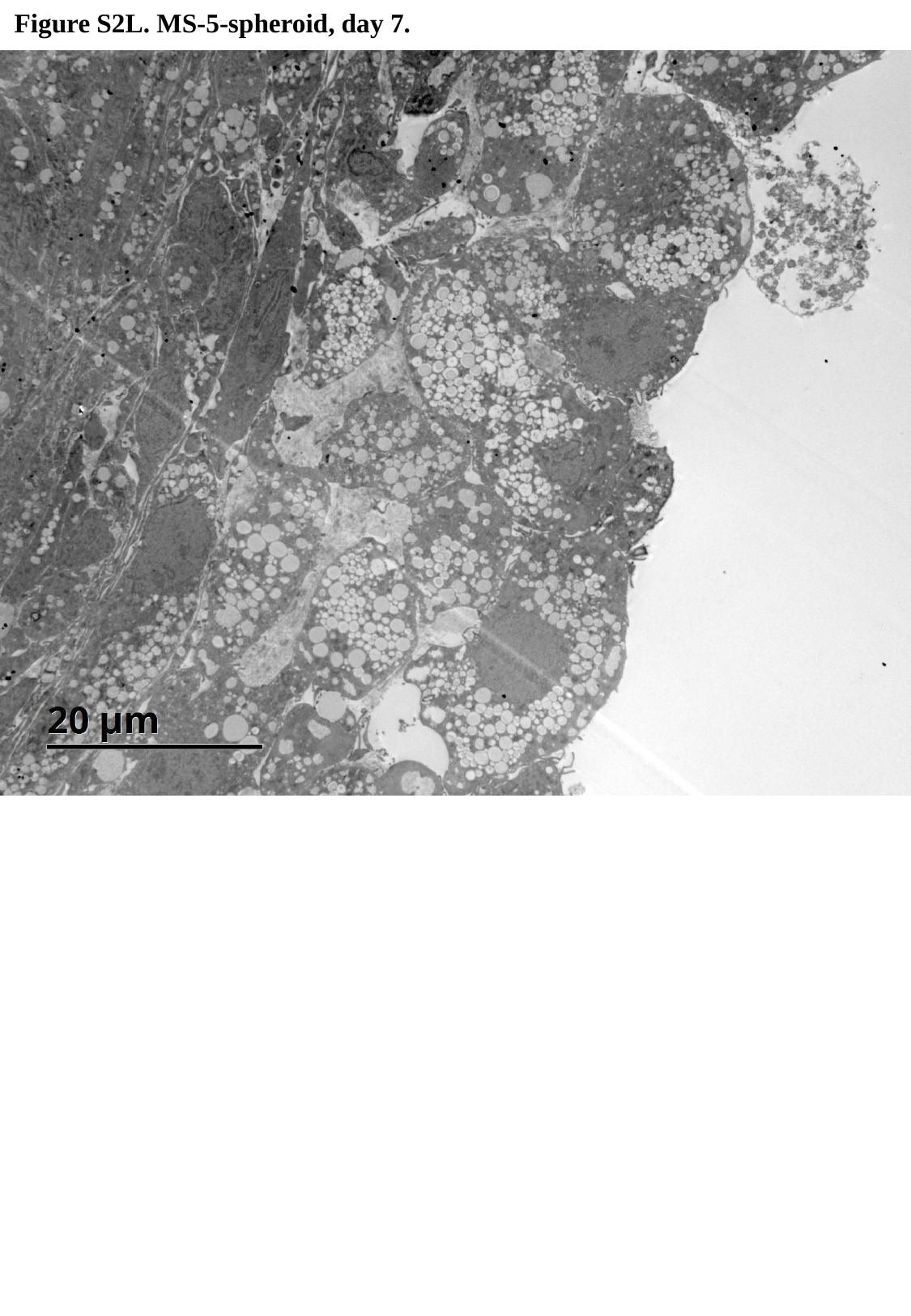

Figure S2L. MS-5-spheroid, day 7.
