# Supplementary material for: A comparative study of the capacity of mesenchymal stromal cell lines to form spheroids
Source: PLoS One. 2020 Jun 2;15(6):e0225485. doi: 10.1371/journal.pone.0225485 (PMC7266346; doi:10.1371/journal.pone.0225485)
Supplement: S1 Table — (DOCX) [file pone.0225485.s008.docx]

**S1 Table. List of primers and probes sequences.**

|  | Probe | Forward primer | Reverse primer |
| --- | --- | --- | --- |
| *ACTB* | gctggaag | attggcaatgagcggttc | cgtggatgccacaggact |
| *BGLAP* | cctggagc | ggcgctacctgtatcaatgg | tcagccaactcgtcacagtc |
| *CAT* | tgctggag | cgcagttcggttctccac | gggtcccgaactgtgtca |
| *EF1A* | ctgctggg | gaaaaatgcttttcgcgatct | atgggaggtcaggcacagt |
| *GAPDH* | tggggaag | agccacatcgctcagacac | gcccaatacgaccaaatcc |
| *GLRX* | ggtggctg | ggcttctggaatttgtcgat | tgcatccgcctatacaatctt |
| *GLRX2 (1)* | ctccatcc | gtggcactcgctggaatc | cgtcgctaaattctccaaagat |
| *GLRX2 (2)* | ccgccgcc | gctggtttggagcaggag | ccaaagatgatgatgtattgctct |
| *GLRX3* | tggtggaa | tcctcaagaaccacgctgt | tgagaagatatcaaaactgctaaactg |
| *GLRX5* | tgctggag | gtgataactggggcgttgtt | actcaggcatgcacagca |
| *GPX1 (1)* | ccaccacc | caaccagtttgggcatcag | gttcacctcgcacttctcg |
| *GPX1 (2)* | ctcctcct | cccttgtttgtggttagaacg | gagagaagggcagctagaacc |
| *GPX3* | aggtggag | cagagatccttcctaccctcaa | ccctttctcaaagagctgga |
| *GPX4* | tggggcag | tacggacccatggaggag | ccacacacttgtggagctagaa |
| *GPX7* | ggaaggag | ccatcctgccttcaagtacc | ttccatctggggctactagg |
| *GSR* | gctggaag | tgccagcttaggaataaccag | cctgcaccaacaatgacg |
| *HIF1A* | ccagccgc | aacctgatgctttaactttgctg | tggtcatcagtttctgtgtcg |
| *NANOG* | cttcctcc | atgcctcacacggagactgt | agggctgtcctgaataagca |
| *OCT4* | ctcctccc | tgcctgcccttctaggaat | caaaaccctggcacaaact |
| *PRDX1* | ccagccag | cactgacaaacatggggaagt | tttgctcttttggacatcagg |
| *PRDX2 (1)* | tggggaag | gccttccagtacacagacgag | gttgggcttaatcgtgtcact |
| *PRDX2 (3)* | cagcctcc | gcaactcagatgcaactctatctact | tgaactggagtttccatcttcat |
| *PRDX3* | ggaagcag | ctggacaccggattctccta | gggtgatctactgatttaccttctg |
| *PRDX4* | actgggaa | gcacctaagcaaagcgaaga | aaattctccatcgatcacagc |
| *PRDX5 (1-3)* | ggaaggag | tcctggctgatcccactg | atgccatcctgtaccaccat |
| *PRDX5 (2)* | ggaaggag | cacccctggatgttccaa | ggacaccagcgaatcatctagt |
| *PRDX6* | cctggagc | caatagacagtgttgaggaccatc | tttctgtgggctcttcacaa |
| *RPL13A* | ccagccgc | caagcggatgaacaccaac | tgtggggcagcatacctc |
| *SOD1* | tggggaag | gcatcatcaatttcgagcag | caggccttcagtcagtcctt |
| *SOD2* | ctgctggg | tccactgcaaggaacaacag | taagcgtgctcccacacat |
| *SOX2* | tcctcttct | ttgctgcctctttaagactagga | ctggggctcaaacttctctc |
| *TXN* | ggctgctg | ttacagccgctcgtcaga | ggcttcctgaaaagcagtctt |
| *TXN2* | ggccccag | gagacaccagtggttgtgga | gcttggccaccatcttctc |
| *VEGFA* | tggtggag | cctccgaaaccatgaacttt | atgattctgccctcctcctt |
